# Short-term plasticity as ‘energetic memory’ of ion channel components of action potential

**DOI:** 10.1098/rsos.231420

**Published:** 2024-06-05

**Authors:** Yuval Ben Abu, Ira Wolfson

**Affiliations:** ^1^ Physics Unit, Sapir Academic College, Sderot, Hof Ashkelon 79165, Israel; ^2^ Department of Physics, Clarendon Laboratory, University of Oxford, Oxford OX1 3PU, UK; ^3^ Department of Physics, International School for Advanced Studies (SISSA), Data Science Excellence Group, Via Bonomea 265, Trieste 34136, Italy

**Keywords:** ion channels, action potential, short-term plasticity

## Abstract

Information transfer in the nervous system is traditionally understood by the transmission of action potentials along neuronal dendrites, with ion channels in the membrane as the basic unit operator for their creation and propagation. We present here a new model for the multiphysics behaviour of ion channels and the action potential dynamics in nervous and other signal-transmitting systems. This model is based on the long-term suppression of an action potential as a response to mechanical input. While other models focus on electrical aspects of the action potential, an increasing body of experiments highlights its electro-mechanical nature and points in particular towards an alteration of the action potential when subjected to a mechanical input. Here, we propose a new phenomenological framework able to capture the mechanical aspect of ion channel dynamics and the resulting effect on the overall electrophysiology of the membrane. The model is introduced here through a set of coupled differential equations that describe the system while agreeing with the general findings of the experiments that support an electro-mechanical model. It also confirms that transient quasi-static mechanical loads reversibly affect the amplitude and rate of change of neuronal action potentials, which are smaller and slower under indentation loading conditions. Changes after the loading release are also reversible, albeit on a different time scale.

## 1. Introduction

Action potential (AP) is arguably the cornerstone of all neuronal information processes [1,2]. It is traditionally measured by electrophysiological methods and is understood as an ion-centred electrical signal propagating along the axonal membrane [2,3]. However, a growing body of studies has shown that AP is accompanied by fast and temporary mechanical changes [4–9]. Specifically, some include alterations in the axonal radius [4–6], while others address pressure [7], optical properties [8] and thermodynamic characteristics [1,2,9]. These are believed to play a significant collaborative role in the functionality of the many processes occurring in cells and tissues for which the final effect is often expressed in biochemical and physiological terms [2,9–11].

During AP propagation, membrane potential increases quickly, up to 500 V/s [12], mediated by ion channels, mainly sodium (Na^+^) and potassium (K^+^) [12,13]. The involvement of Na^+^ and K^+^ ion channels as the basic building blocks of AP is well documented. Specifically, the involvement of these channels in physiological processes in many organisms, ranging in complexity from viruses to mammals, has been studied extensively [1,2,13,14]. These processes play an essential role in generating and regulating the electrical activity of cells in the nervous and muscle systems [2,15] and are optimally tuned for rapid information processing [9]. These channels have three main properties: rapid conduction of ions; selectivity to Na^+^ and K^+^ ions; and opening and closing by specific electrical, mechanical or chemical signals [13,16]. K^+^ and Na^+^ channels allow ions to diffuse down the electrochemical gradient across the membrane under physiological conditions [17]. While some ion channels actively change the potential gradient, some allow the passive diffusion of ions down an external potential gradient [18]. Manipulation of these channels has shown clear effects on AP in many aspects [19], of which drug blockers, mutations and toxins are only a partial list [20,21].

Na^+^ channels are integral membrane proteins that belong to the superfamily of cation channels. In excitable cells such as neurons, myocytes and certain types of glial cells, they are responsible for the rising phase of APs [1]. This phase includes a depolarizing wave, and the channel going through three different states is called the resting, active and inactive states [22]. K^+^ channels are the most widely distributed type of ion channels and are found in virtually all living organisms [23]. They form K^+^-selective pores that span cell membranes, are found in most cell types and control a wide variety of cell functions [1].

K^+^ channels conduct K^+^ ions down their electrochemical gradient, both rapidly and selectively [13,15]. The main difference between these two channels, in addition to selectivity and the way they conduct ions, is their size [1,13]. For instance, the larger Na^+^ channels have four repeat domains, each containing six membrane-spanning segments (S1–S6). The highly conserved S4 segment acts as the channel’s voltage sensor. The voltage sensitivity of this channel is owing to positive amino acids located in every third position. When stimulated by a change in transmembrane voltage, this segment moves towards the extracellular side of the cell membrane, allowing the channel to become permeable to ions [1]. In K^+^ channels, the size differs to account for different involvement periods, as well as different roles. However, they are always small in comparison to Na^+^ channels. Voltage-dependent K^+^ channels that mainly drive the AP repolarization phase have four subunits with voltage sensors. Furthermore, some types of K^+^ channels are even smaller than the usual K^+^ channels and have two-pore domains at the same phase [24].

Despite this wealth of experimental evidence regarding APs and their characteristics, e.g. depolarization and repolarization, the physical basis for the mechanical and thermal signals that accompany AP is still little understood [3,10,25]. To the best of our knowledge, no attempt has been made to describe and model quantitatively the molecular and mechanical components of AP as an electrically driven phenomenon incorporating mechanics-driven mechanisms. Here, we describe the energetic molecular basis accompanying AP propagation. As AP propagates, changes in charge separation across the dielectric membrane alter surface forces, in turn affecting the membrane’s geometry. At the same time, the constituent channels of AP undergo mechanical changes.

The existing mathematical descriptions of AP, starting with the pioneering work of Hodgkin and Huxley [2], consistently define the behaviour of ion channels with a phenomenological short-term memory of the channel to account for the refractory period, with a set of self-referential differential equations for the Hodgkin–Huxley (HH) model [2].

However, outside the realms of mechanosensitive ion channels, the direct effect of mechanical manipulation of ion channels on AP has largely been overlooked [20] and has only seen an increase in interest in the past decade or so, mostly in the context of axonal injury, and, more recently, ultrasound neuromodulation [26]. Paradoxically, it was shown over half a century ago that local mechanical changes propagate in the neuronal membrane in phase with AP [6]. These observations suggest that, in addition to AP’s electrical properties, a complex multi-scale electro-mechanical coupling involving all components of the membrane exists.

**Figure 1.**
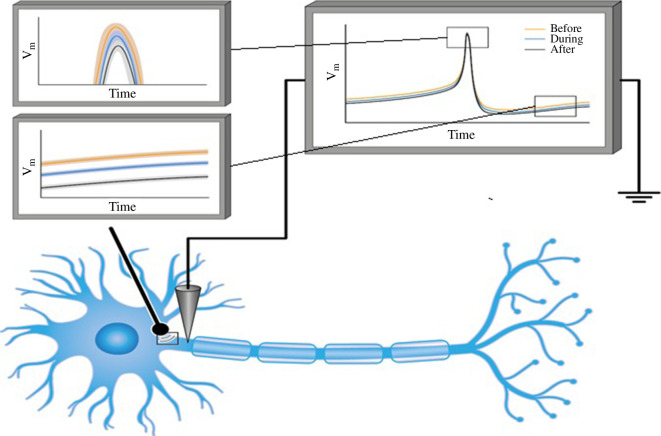
Left: AP response of a patch-clamped individual F11 cell before, during (shaded region) and after 80 s long 3 µm indentation, which covers several APs. Centre: mean and standard deviation of all APs on the left, with peaks in the inset. Right: mean and standard deviation of all AP phase plots (this figure is based on data from [26]).

Additionally, it was shown that beyond the biochemical, physiological or electrophysiological effects of the K^+^ and Na^+^ ion channels on AP, other critical factors affect AP shape, propagation and regulation [3]. In particular, in an experiment performed by Tamayo-Elizalde *et al*. [26], the authors applied external pressure quasi-statically to an ion channel aggregate and studied the changes in AP voltage levels following indentation (see figure 1).

These results indicate that no significant change in the baseline voltage level was detected during quasi-static indentation. However, when the pressure was relieved quasi-statically, a detectable deviation from the AP baseline voltage was observed (see figure 1) [26].

Here, we propose a physically analogous system that simulates longer changes relative to a single AP cycle period, mimicking the system’s long-term self-reference of its previous state (in this case, the indentation stress), and couple the resulting long-term hysteresis to the HH model [2]. Ultimately, we show that the model correctly approximates the behaviour of experimental results found in Tamayo-Elizade *et al*. [27] and other experimental work. It is important to understand that, while we use a physical analogue to understand the phenomenology, the model is simply a ‘next-term approximation’ for the HH model. The amended equations simply account for changes in channel conductivity owing to the physical morphology changes of the channels from either external or internal forces.

## 2. Theoretical framework

The normal operation of an ion channel is to expand and contract alternately, affecting the channel’s ion throughput. We assume here that the channel is a normally neutral channel, such that a baseline amount of energy is invested in the channel’s closure mechanism. Applying more energy than the baseline results in further deformation and tighter closure of the channel, thus diminishing the ion throughput. Note that this assumption does not account for the mechanisms involved in the functioning of specifically mechanosensitive^1^ ion channels. A reduction in the energy absorption below the baseline will result in looser closure and a larger ion current.

The nature of the external mechanical loading has not been specified, i.e. whether it is a compressive force in the membrane (thus intuitively closing the channel in agreement with the following description), or it is similar to secondary mechanisms leading to an effective closure of the channel (such as stretching of the membrane leading to inverse mechanical effects on the channel through the action of the cytoskeleton and/or extracellular matrix, out-of-plane forces with respect to the membrane, bending or shearing forces). Here, and subsequently, we thus refer to ‘external mechanical loading’ as any external mechanical loading interfering with the channel to impede its normal opening.

Let $\varepsilon_{0}$ be the baseline energy associated with the membrane’s resting state and $S_{0}$ the channel’s cross-sectional area corresponding to the ion current with baseline energy $\varepsilon_{0}$ . Furthermore, for our purposes, we can assume without loss of generality that $r_{0}$ is the effective radius of $S_{0}$ , such that:


(2.1) $$r_{0}^{2}\propto S_{0}=f\left(\varepsilon_{0}\right),$$

where f:ε→S is a function that maps the cross-sectional area response to the energy absorption in the membrane. With these conventions, higher energy absorption leads to constriction (up to and including closure), such that:


(2.2) $$\varepsilon_{constricted}>\varepsilon_{0}>\varepsilon_{dilated\;}$$

and


(2.3) $$S_{constricted}=f\left(\varepsilon_{constricted}\right)<f\left(\varepsilon_{0}\right)<f\left(\varepsilon_{dilated}\right)=S_{dilated}.$$

When an external force is applied to close the channel mechanically, a smaller energy absorption is required to achieve full closure. Thus, noting $S_{closed}=S_{0}-\Delta S=S^{\prime }-\Delta S^{\prime }$ , where $S^{`}$ is the new cross-sectional baseline owing to the external force, ΔS and $\Delta S^{`}$ are the relative changes from the baseline to the closed state, without and with the external force, respectively, it follows that:


(2.4) $$S^{\prime }<S_{0}⟹\Delta S^{\prime }<\Delta S.$$

**Figure 2.**
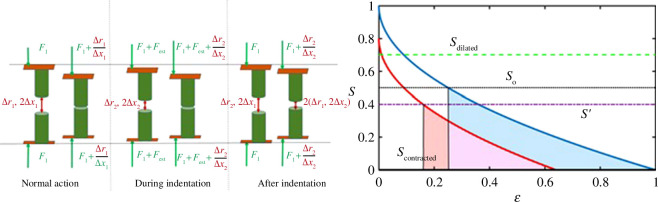
The three stages of mechanical loading are presented in the left panel. In normal action (with normal physiological force F_1_ arising, for example, from the surrounding lipid layers), the channel is fully open or fully closed with an increase in energy $\Delta \varepsilon_{1}$ (blue curve on the graph on the right). During mechanical loading, the channel is constricted in the open position, such that it is already partially closed, and only $\Delta \varepsilon_{2}$ is required for full closure. After the indentation removal, owing to the lack of available energy or ‘long-term hysteresis’ while the channel can fully open, it can no longer fully close (red curve on the graph on the right). In the area versus energy plot (right panel), the blue line corresponds to the case without an external force, while the red line is when the external force is applied. An external force that constricts the initial channel’s cross-section from $S_{0}$ (black dots) to $S^{\prime }$ (purple dash-dot) results in a lower energy absorption needed to achieve full closure (taken arbitrarily here to be S=0 in the graph where units are also arbitrarily chosen for illustration). This is true for any strongly monotonically decreasing function. All scales are arbitrary.

Thus, a smaller amount of additional energy absorption is needed to fully close the channel. This is shown schematically in figure 2, where the external force moves the cross-section to the energy function from the normal trajectory to a different one, resulting in a smaller energy absorption needed to achieve the same fully constricted, or closed, cross-section:


(2.5) $$\Delta \varepsilon^{,}<\Delta \varepsilon ,$$

where Δε and $\Delta \varepsilon^{,}$ are the energy provided to the system without and with external force, respectively, to reach its closed state.

Since, a quantity Δε of energy is available under normal circumstances, investing $\Delta \varepsilon^{\prime }$ is possible and the channel fully contracts. A common heuristic in properly working biological systems is feedback mechanisms that are applied to ensure efficiency [28]. By this heuristic, we suggest that the system now adapts to allocate only $\Delta \varepsilon^{\prime }$ amount of energy towards channel closure requirements, while the remainder of the energy is reallocated to other processes.

Thus, after the external mechanical loading is relieved, the channel sub-system now has insufficient energy to fully contract, and the throughput in the fully contracted state is higher than normal (see figure 2, left panel, after mechanical loading). Note that the system can possibly re-adapt later to this new state and eventually reallocate the necessary energy to fully contract the channel. However, these adaptation and re-adaptation processes occur over a time frame longer than that of a few AP cycles. For instance, in figure 1, we see that the baseline voltage remains hyperpolarized at least hundreds of seconds after the indentation is removed, whereas the AP timeframe is typically a few milliseconds, with an upper bound of a few seconds [28,29].

Furthermore, if we assume that a similar external indentation is applied whereby the effective radius of two channels of different radii is decreased by the same amount Δr , the shift in cross-section would be more pronounced for the narrower channel. This is evident by:


(2.6) $$r_{1}^{2}\propto S_{1}>S_{2}\propto r_{2}^{2}$$

(2.7) $$\frac{\Delta S_{1}}{S_{1}}\propto \left(\frac{\Delta r}{r_{1}}\right)<\left(\frac{\Delta r}{r_{2}}\right)\propto \frac{\Delta S_{2}}{S_{2}}$$

in which the index 1 denotes the wider channel and index 2 the narrower one.

This means that the difference in energy requirement will be more pronounced for the narrower channel. So, after the external force is removed, a larger energy deficit for the narrower channel means a higher (positive) throughput change in the narrower K^+^ channels, especially the K_2P_.

## 3. Methods

The following computational analysis and simulation were done using MATLAB [a] and the computational methods therein.

Where functions are known, they are implemented as anonymous functions in MATLAB (using the @(x) notation). External pulses of current input are simulated using the Heaviside step function with the appropriate timing. The indentation of the channel’s membrane s simulated using a hyperbolic tangent function (tanh) starting at *t* = 100 s and ending at *t* = 180 s. The width of the tanh function is *d* = 10 s. Thus, the indentation function used is the following:


$$D=\left(tanh\left(\frac{t-100}{10}\right)-tanh\left(-\frac{t-180}{10}\right)\right).$$

The reasoning behind the rather slow indentation is the requirement of adiabaticity, i.e. a very slow injection of energy into the system by external mechanical means. The numerical integration was done using the so-called Runge–Kutta 45 method, which since 1997 has been implemented using a Dormand-Prince pair. (The code itself is publicly available at the git depository of I.W.) [30–32].

## 4. The model: mechanical analogue of a system with hysteresis

**Figure 3.**
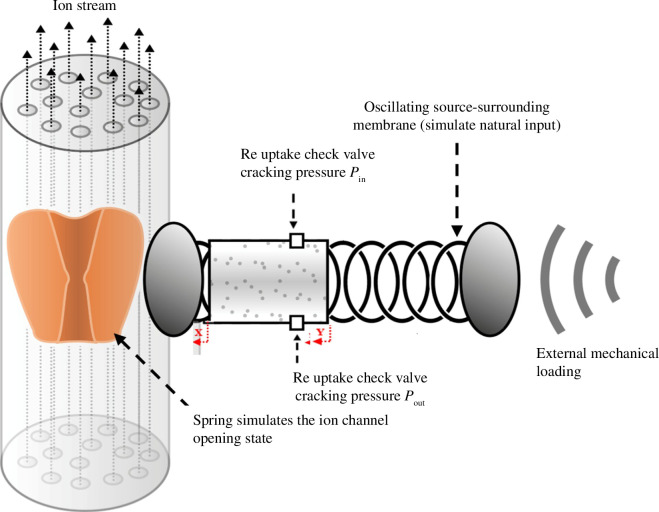
A mechanical system that displays long-term hysteresis can be interpreted as memory. The system comprises two springs separated by an ideal gas-filled piston. The piston has lower- and upper-pressure thresholds, Pin and Pout, respectively. When the internal pressure is above (below) the higher (lower) threshold, the output (input) check valve cracks, allowing the internal pressure to start equilibrating with the environment. X and Y represent the displacements of the right- and left-hand sides, respectively, of the piston with respect to the resting state (positive being compressive with respect to the ion channel spring).

In the following section, we consider an idealized composite spring-piston model to simulate a system that sinks into a lower energetic state upon the application of external mechanical loading, while conserving some reference to the acquired change akin to long-term hysteresis. That is, when an external force, for instance ultrasound (US) pulse, is applied to the system, the system initially goes to a higher energetic state. This higher energetic state is followed by an energetic state lower than the original, thereby allowing enhanced ionic current throughput. Physically, this is similar to a system in a meta-stable state. The system can reside in that meta-stable state for a prolonged period of time. However, upon introducing additional energy, the system will soon fall into the stable state. Finally, after a refractory period, the system is reset into a meta-stable state by, for example, coupling to some thermal bath. The model comprises two springs separated by an ideal gas-filled piston (see figure 3). The left spring simulates the ion channels *per se*, while the piston reproduces the long-term suppression of the system, and the spring on the right represents the rigidity of the surrounding membrane. The piston has two ‘valves’. When the internal pressure in the piston rises above the higher-pressure threshold, the higher-pressure valve opens and releases gas until the internal pressure sinks back below the higher-pressure threshold. Conversely, when the internal pressure sinks below a lower-pressure threshold, the lower-pressure valve opens to introduce gas into the system until the internal pressure rises back above the lower-pressure threshold.

The external load is applied on the right-hand side of the system, and the system’s response is measured at the right-hand side of the left spring. Thus, when the external load drives the overall system to compress, the right-hand side spring is compressed. The pressure in the piston then rises, compressing in turn the left spring. If enough load is applied, some gas exits the piston, effectively shifting the system’s equilibrium point.

In this manner, the system ‘remembers’ that it has previously been compressed. It is possible to implement a dynamic recovery by allowing the lower-threshold valve to activate. The difference between the outward and inward flow rates at the valves controls the rate of recovery and may be considered quasi-static. The system is shown in figure 3.

### 4.1. Equations of motion for the mechanical system

In the following section, we describe the coupled differential equations of motion for the system. The pressure P inside the cylinder obeys the ideal gas law and can be calculated under isothermal conditions by:


(4.1) $$P=\frac{C}{Vol},$$

where C is some constant that represents the ideal gas constant multiplied by the constant temperature and Vol is the volume of gas in the piston. Alternatively, note that it is possible to use the relation $P\;{\left(Vol\right)}^{\gamma }=C$ for isentropic conditions. In this case, the equations below can be modified trivially to account for the change.

The system of differential equations is given by:


(4.2) $$\left\{ \begin{array}{l} \overset{˙}{P}=\;-\frac{P}{Vol}A\left(\overset{˙}{X}-\overset{˙}{Y}\right)-RH_{step}\left(P-P_{out}\right)\times \left[P-P_{out}\right]+\tilde{R}H_{step}\left(P_{in}-P\right)\times \left[P_{in}-P\right]\\ \overset{¨}{Y}=\frac{-A\left(P-P_{0}\right)-k_{u}\left(Y-D\left(t\right)\right)}{M_{u}}\\ \overset{¨}{X}=\frac{A\left(P-P_{0}\right)-k_{d}\left(X\right)}{M_{d}}, \end{array}\right.$$

where A is the cross-sectional area of the piston, $P_{0}$ is the initial pressure at equilibrium in the piston, $P_{out}$ is the upper-pressure threshold, $P_{in}$ is the lower-pressure threshold, $k_{u}$ and $k_{d}$ are the spring constants, $H_{step}\left(Z\right)$ is the Heaviside step function such that $H_{step}\left(Z\right)$ =1 for Z§amp;gt;0 and $H_{step}\left(Z\right)=0$ otherwise, and $M_{u}$ and $M_{d}$ are the piston terminal masses at the upper and lower ends, respectively. X and Y are the positions/displacements of the right- and left-hand sides, respectively, of the piston as indicated in figure 3. Finally, $D\left(t\right)$ is the external displacement introduced by a cantilever, for example, or any other indenter. The default value and units of these quantities are presented in table 1.

**Table 1.** Additional parameters for the coupled system. All quantities are dimensionful and are measured in the same units as their reference quantities. For instance, $r_{0,i}$ is measured in the same units as the channels (i.e. µm in this case). All of the piston parameters can be viewed as dimensionless, with a dimensional constant that scale X and Y , the result being measured in µm. The piston measurements such as its cross-section or rest length are superfluous, as for every choice of *A* and *L*; we can find a $P_{0}$ and a set of $K_{i},M_{i}$ such that one of the $\delta_{i}$ S is calibrated to some value. The only important constant in this system is the ratio of channel radii.

$r_{0,K}$	K-channel rest radius	50 µm	$M_{i}$	masses that both plates are set to	1 kg	R	rate of gas leakage from the piston	1 [Pa/s]
$r_{o,Na}$	Na-channel rest radius	5 µm	*L*	piston length	1 mm	$\tilde{R}$	rate of gas intake	0
$r_{0,leak}$	leak K- channel rest radius	0.5 µm	A	piston cross- sectional area	1 $mm^{2}$	$P_{out}$	upper crack valve pressure	$P_{0}\left(1+10^{-3}\right)$ [Pa]
$K_{i}$	spring constants	1 N/m	$P_{0}$	initial gas pressure in the piston	30 Pa	$P_{in}$	lower crack valve pressure	$P_{0}\left(1+10^{-3}\right)$ [Pa]

Vol is calculated according to:


(4.3) Vol=A(L−X+Y),

where L is the rest length of the piston. The second term of the right-hand side of the first equation in equation (4.2) is associated with the upper threshold pressure valve.

When $P>P_{out}$ , this term removes gas from the piston at rate R , thus decreasing the internal pressure. Conversely, the third term is associated with the re-uptake crack valve, such that when $P_{in}>P,$ gas is introduced into the piston at rate $\tilde{R}$ .

**Figure 4.**
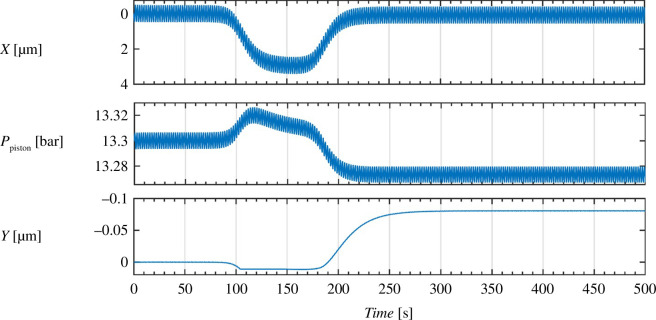
A simulation of the piston system, with an initial piston loading pressure of 13.3 bars, upper threshold of 13.31 bars and lower threshold of 13.29 bars. In this system, we also implemented a lower ‘dead-point’ for X such that it cannot move over 0.02 µm in the downwards direction. This is done to simulate a limit for deformation, meaning that the overall position of the channel(s) is fixed. The system begins at a baseline of 0 external displacement, undergoes a quasi-static period of displacement at t∈(100,180)s , up to a maximum of 3 µm. The displacement then subsides to zero quasi-statically at t∈(180,500) s. It is shown clearly that the system ‘remembers’ having been pressed.

In figure 4, the system’s response to an external displacement input is shown for an arbitrary oscillatory input of 0.9 µm with a frequency of 3 Hz to show the quasi-static of the system. The system ‘remembers’ having been pressed, moreover, the high-frequency input is suppressed in the ion channels. In this simulation, we set the outflow rate *R* = 1.3 μbar/s to set a timescale that is much longer than the timescale of the single AP cycle. The inflow rate is set to $\tilde{R}=0$ , but re-uptake can easily be reintroduced. Note that this is not yet the simulation of the complete system.

### 4.2. Long-term mechanical hysteresis-modified Hodgkin–Huxley model

The HH model retains some short memory of its previous state, as evidenced by the $\left(\frac{dV}{dt},V\right)$ hysteresis-type phase space of a single AP cycle changing after indentation (see figure 1). Here, we couple the usual HH model differential equations with the spring-piston mechanism introduced above to incorporate a mechanically based long-term self-reference to its deformation state. This long-term memory-like mechanism is hinted at when one considers the experimental results in [27], and the change in single AP hysteresis loops between pre-, mid- and post-indentation results.

The HH model differential equations are given by:


(4.4) $$\left\{ \begin{array}{l} \overset{˙}{n}=a_{n}\left(V\right)\cdot \left(1-n\right)-b_{n}\left(V\right)\cdot n\\ \overset{˙}{m}=a_{m}\left(V\right)\cdot \left(1-m\right)-b_{m}\left(V\right)\cdot m\\ \overset{˙}{h}=a_{h}\left(V\right)\cdot \left(1-h\right)-b_{h}\left(V\right)\cdot h, \end{array}\right.$$

where n,m and h are the gating functions and the functions $a_{n}$ , $a_{m}$ , $a_{h}$ , $b_{n}$ , $b_{m}$ and $b_{h}$ are given in table 2. These gating functions denote the probability of a specific channel activation and enable the ion current flow. Usually, n is associated with the potassium channel, m with the sodium channel and h with the sodium channel inactivation.

**Table 2.** HH parameter initial values and functions. The external activation current $I_{ext}$ is given by square pulses of 12.5 ms with 12.5 ms spacing. Here, the resting potential is taken to be −65 mV, and voltage units are in mV. The external force on the piston’s pressure terminal is such that there is a 3 µm maximal indentation for 80 ms. In this model, $c_{m}$ is set to 1 nF and conductivity is measured in nano Siemens (nS).

$a_{n}$	$\frac{0.01\left(V+55\right)}{1-e^{-\frac{V+55}{10}}}$	$b_{n}$	$0.125e^{-\left(\frac{V+65}{80}\right)}$	${\tilde{g}}_{K}$	36 nS
$a_{m}$	$\frac{0.1\left(V+40\right)}{1-e^{-\frac{V+40}{10}}}$	$b_{m}$	$4e^{-\left(\frac{V+65}{18}\right)}$	${\tilde{g}}_{Na}$	120 nS
$a_{h}$	$0.07e^{-\left(\frac{V+65}{20}\right)}$	$b_{h}$	$\frac{1}{1+e^{-\left(\frac{V+35}{10}\right)\;}}$	${\tilde{g}}_{l}$	0.3 nS
$n_{0}$	0.32	$m_{0}$	0.05	$h_{0}$	0.6
$E_{Na}$	50 mV	$E_{k}$	−77 mV	$E_{l}$	−54.387 mV

The membrane potential evolution is then dictated by:


(4.5) $$\overset{˙}{V}=\frac{I_{ext}-n^{4}{\tilde{g}}_{K}\left(V-E_{K}\right)-m^{3}h{\tilde{g}}_{Na}\left(V-E_{Na}\right)-{\tilde{g}}_{l}\left(V-E_{l}\right)}{C_{m}},$$

where ${\tilde{g}}_{K}$ , ${\tilde{g}}_{Na}$ , ${\tilde{g}}_{l}$ , $E_{K}$ , $E_{Na}$ and $E_{l}$ are the reference conductivities and reversal potentials for K^+^, Na^+^ and current loss, respectively, $C_{m}$ is the membrane capacitance and $I_{ext}$ is an external current that is potentially applied (for AP triggering) (see table 2).

More details on the model are available in [2]. , which was canonized and appears in most current textbooks, cf. [29].

### 4.3. Coupling the piston’s displacement with the channel conductivity

To couple the spring-piston system to the HH model, we propose that the external mechanical load changes the channel’s morphology following the rules explained above, in turn altering conductance. Indeed, while the channel can normally accommodate a certain maximal value of ion current, the application of an external mechanical load constricts the channel down to a certain value. After the external load is removed, the membrane wall cannot constrict the current as it normally does. Thus, the maximal ion current value increases. This can be simulated by changing the value of conductivity ${\tilde{g}}_{i}$ for the different channels, each denoted by i , such that:


(4.6) $${\tilde{g}}_{i}\to \left(1-\delta_{i}\right){\tilde{g}}_{i}$$

where $\delta_{i}$ is an increasing monotonous function of X (the piston’s lower plate displacement) and takes different values for each channel: the more compressed the channel is, the less its conductance. The physical width of the channel can explain the differences in dynamics between different channels. Thus, the amount of energy reduction needed to close the channel and subsequently, the amount of energy eventually lacking for normal closure of the channel differs. In the following equation, we assume that the function $\delta_{i}\left(X\right)$ is simply given by:


(4.7) $$\delta_{i}\left(X\right)=\frac{X-X_{0}}{r_{0,i}}$$

where $r_{0}$ is the channel rest radius and X is informed by the previous mechanical model. We set $X_{0}=0$ .

The spring-piston and HH model coupling is thus weak in the sense that the spring-piston system affects the HH model, but there is no significant feedback from the HH model to the spring-piston system.

Again, we stress that while we use a physical analogue implementation, the equations account for a linear correction to the conductivity of the channels owing to either external (cf. indentation, shear, stretching, etc.) or internal physical changes. Further analysis and experiments are called for to tune the model. The long-term hysteresis is achieved owing to a mismatch in time scales between the conductivity changes and the gating dynamics.

With this adaptation, equation (4.5) becomes:


(4.8) $$\overset{˙}{V}=\frac{I_{ext}-n^{4}\left(1-\delta_{k}\right){\tilde{g}}_{K}\left(V-E_{K}\right)-m^{3}h\left(1-\delta_{Na}\right){\tilde{g}}_{Na}\left(V-E_{Na}\right)-\left(1-\delta_{l}\right){\tilde{g}}_{l}\left(V-E_{l}\right)}{C_{m}}.$$

**Figure 5.**
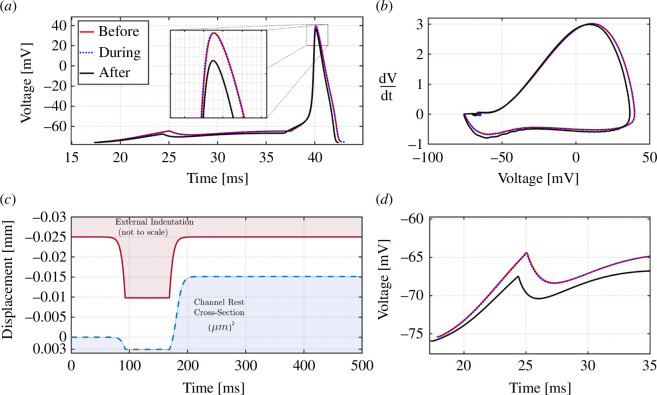
A coupled spring-piston and HH model reveals a mechanism in which the system remembers previous strains. In (*a*), we see the system response to the external displacement (*c*, red) by the membrane wall (*c*, blue) slightly collapsing and constricting the ion current flow. As a result, the baseline AP (red line) is changed to an altered AP (black line) with slightly different characteristics. The phase-space diagram (*b*) shows that when the channels are constricted (blue dots), the AP changes only slightly, but when external pressure is relieved, the dV/dt versus V-cycle (or AP dynamics) is visibly changed. Finally, the refractory period increases after indentation and relief (*d*).

Figure 5 shows the results of this simulation of coupled systems for the following parameters and loading conditions. The parameters are the same as in table 2. However, new parameters are introduced in coupling the systems. The ratio between the rest radii of the channels is 50 : 5 : 0.5 for the K-channel, Na-channel and leak channel, respectively. Other constants parametrize the piston, and are thus less important. All parameters are given in table 1. The input current is given by:


(4.9) $$\sum_{i=1}^{7}7\times \left(H\left(t-\left(2i-1\right)\times 12.5\right)-\;H\left(t-2i\times 12.5\right)\right)[pA].$$

Additionally, the code is made publicly available at https://github.com/beastraban/HH_With_Piston.git.

We see that the system responds to external mechanical loading much as one would expect, and the resulting AP under pressure is only slightly changed. However, when the load is relieved, the membrane wall loses its ability to close properly and efficiently, thus the AP shape is visibly changed owing to increased ion current flow in each channel.

**Figure 6.**
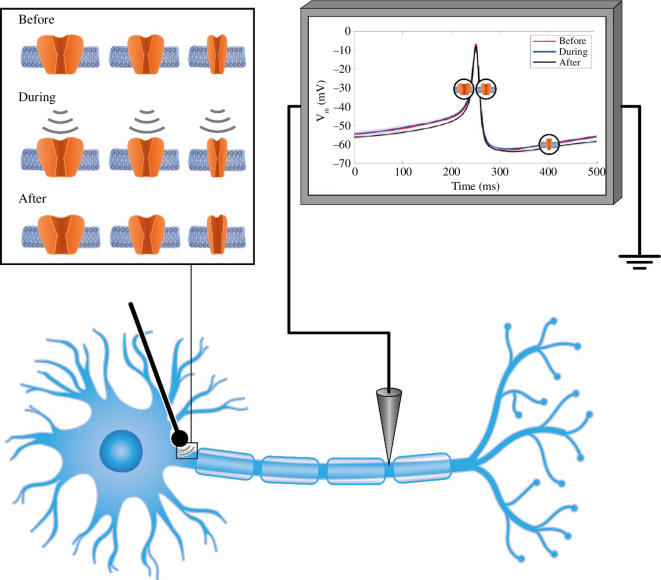
Nanonindentor effect on molecular components of action potential. The upper right side represents the recording nanoindentor effect (lower left side of the neuron). The upper left side shows the effect on Na^+^ (left column), K_v_ (centre column) and K_2P_ (right column) channel before, during and after the nanoindentor ‘was placed’ on the neuron. It can be seen that the channels are more open after the nanoindentor release in comparison to before the nanoindentor was placed.

A diagram of the model and simulation is shown in figure 6, where we also provide a visual of what we posit happens to the ion channels.

## 5. Discussion

Many diverse studies have shown that a mechanical displacement of the axonal membrane accompanies the electrical pulse defining AP [25]. While measuring and understanding the electrical component of AP has been the focus of most experimental and theoretical efforts, a large number of experimental studies have shown that AP is accompanied by rapid and temporary mechanical changes [25]. These include changes in axonal radius, pressure, optical properties, electricity field change of ion channels, etc.

Although the vast majority of theoretical and experimental efforts in neuroscience aim at understanding the electrical component of AP, some are trying to understand the electro-mechanical component of AP. For instance, Schewe *et al*. [33] showed that some K_2P_ channels operate without voltage sensing and display mechanical morphology following a change in the electricity field in the pore channel [33]. 30 On the molecular level, Kim and Chang [34] focused on patch clamp technology for focused ultrasonic neuromodulation [34].

The most basic ion channels, Na^+^ and K^+^, play distinct roles in AP [16] across two main phases: the depolarization phase, driven mainly by Na^+^ channel input; and the repolarization phase, driven mainly by voltage-gated K^+^ channels (K_v_) and leak K^+^ channels (K_2P_). Members of the two K^+^ channel families, K_2P_ and K_v_, belong to the dimeric two-pore domain and tetrameric K^+^ channel varieties, respectively. K_2P_ channels are open at all membrane voltages [16] and are therefore involved in determining the threshold for AP generation and controlling AP repolarization and firing frequency [16,31]. K_v_ channels, in contrast, are closed at negative membrane voltages and are only open upon membrane depolarization [13,16]. Voltage-gated Na^+^ channels play an essential role in initiating and propagating APs in neurons and other electrically excitable cells such as myocytes and endocrine cells [32,35]. When a few millivolts depolarize the cell membrane, sodium channels activate and inactivate within milliseconds. Influx of sodium ions through the integral membrane proteins comprising the channel depolarizes the membrane further and initiates the rising phase of the AP. The voltage-gated Na^+^ channel is a large, multimeric complex composed of an α subunit and one or more smaller β subunits [36].

In this manuscript, we developed a new theory that suggests two plausible mechanisms: (i) the mechanism of ion channel conductivity is sensitive to outside mechanical inputs and (ii) as a result, it may be sensitive to sound-wave input, thus explaining some of the apparent efficacies of ultrasound neuromodulation [24]. Another aspect of this model is the relatively long-term hysteresis or ‘energy memory’ of the AP and ion channels. This would provide a possible explanation for the previously marginally explained phenomenon of baseline voltage changes in AP recording experiments [26]. Furthermore, the ratio between K^+^ channel and Na^+^ channel changes may give us a valuable hint as to the physical size and composition of the K^+^ channel and could very well represent a method of probing these channels in further detail than is currently available.

When considering AP’s refractory period shown in figure 1, it is difficult not to consider the magnitude of the effect of the size of the ion channels, especially on the K_2P_ channels. This type of channel has been identiﬁed in many sites throughout the nervous system and has been shown to exhibit unique electrophysiological and pharmacological properties [35,37,38]. The main role of these channels is the rapid and efficient passage of K^+^ ions, giving rise to the refractory period between one AP and another. Some evidence has suggested that the main channels involved in the AP depolarization and repolarization phases are the K_2P_ channels [39,40]. Generally, the Na^+^ channel’s size is larger than that of the K^+^ channels, and the K_2P_ channels are even smaller than the voltage-gated or ligand-dependent potassium K^+^ channels. K_2P_ channels have four subunits that are repeated four times compared to six in the voltage-dependent or ligand-dependent K^+^ channels. The model presented here shows that channel size has crucial importance in the repolarization phase after applying an external force to a neuron.

Interestingly, applying pressure on the channels altered the AP’s refractory period, which involves mainly K_2P_ channels, the smallest channels in this process. Owing to their size, pressure on the neuron will have a significant effect on these channels compared to the larger K^+^ channels. In the next pulse, the neuron, together with its ion channels, has a mechanical memory of the optimal energy needed to close the channels. Thus, pressure on the neuron leads to the closure of the channels relative to the channel’s type: Na^+^ channels have a lesser effect compared to different types of K^+^ or K_2P_ channels. However, according to figure 1, the channels adapt to the energy amount needed to close them as a result of the last pulse. Applying pressure to the K^+^ channels, especially the small K_2P_ channels, will somehow close the channel but not completely; the energy gap needed to close them will be adapted during the next pulse. Thus, at the next pulse, the energy needed to close the channels completely is lower than usual and the open conformation of the channels will be more stable, thus explaining the hyperpolarized membrane voltage of the AP shown in figure 1. Over time, these channels return to normal activity owing to an increase in energy amount.

A similar phenomenon occurs in the Na^+^ channels. The difference is that the energy needed for closure is much lower; therefore, the differences between the two occurrences, as shown in figures 3 and 6, are smaller than the one presented in figure 1. Applying this force using a nanoindentor on the neuron would affect different phases of the AP (figures 1–3 and 6). However, the main differences occur in the periods before the AP and at the end of the repolarization phase [38–41]. Interestingly, the main channels that exhibit high activity during these AP stages are the leak channels, the K_2P_ channels—the smallest and most susceptible channels to pressure effects [42]. Figure 6 presents the effect of the nanoindentor on the AP and its components: the Na^+^ and K^+^ channels.

As with all models, additional probing and testing will either disprove or support it. However, this model can at least explain some of our notions and observations of a wide variety of phenomena. From ultrasound pain treatment, through the efficacy of treatments by applying local pressure (i.e. massage therapy), up to and including some of the adaptive aspects of organisms to pressure differentials.

## Data Availability

Data and relevant code for this research work are stored in GitHub [42] and have been archived within the Zenodo repository [43].

## Footnotes

^1^
While we implicitly argue that all channels are mechanosensitive by virtue of mechano-electrophysiological coupling, we refer to “mechanosensitive ion channels” as ion channels whose function is to specifically open or close when subjected to a specific mechanical loading.
